# Single nucleotide polymorphisms within the Wnt pathway predict the risk of bone metastasis in patients with non-small cell lung cancer

**DOI:** 10.18632/aging.103207

**Published:** 2020-05-26

**Authors:** Yiquan Xu, Hongru Li, Lihong Weng, Yanqin Qiu, Junqiong Zheng, Huaqiang He, Dongmei Zheng, Junfan Pan, Fan Wu, Yusheng Chen

**Affiliations:** 1 Shengli Clinical Medical College of Fujian Medical University, Fuzhou 350001, China; 2 Department of Respiratory Medicine and Critical Care Medicine, Fujian Provincial Hospital, Fuzhou 350001, China; 3 Fujian Provincial Researching Laboratory of Respiratory Diseases, Fuzhou 350001, China; 4 Department of Medical Oncology, Longyan First Hospital Affiliated to Fujian Medical University, Longyan 364000, China

**Keywords:** Wnt signaling pathway, polymorphisms, bone metastasis, risk, biomarker

## Abstract

The Wingless-type (Wnt) signaling pathway plays an important role in the development and progression of cancer. This study aimed to evaluate the relationship between single nucleotide polymorphisms (SNPs) in the Wnt pathway and the risk of bone metastasis in patients with non-small cell lung cancer (NSCLC). We collected 500 blood samples from patients with NSCLC and genotyped eight SNPs from four core genes (WNT2, AXIN1, CTNNB1 and APC) present within the WNT pathway. Moreover, we assessed the potential relationship of these genes with bone metastasis development. Our results showed that the AC/AA genotype of CTNNB1: rs1880481 was associated with a decreased risk of bone metastasis. Polymorphisms with an HR of < 1 had a cumulative protective impact on the risk of bone metastasis. Furthermore, patients with the AC/AA genotype of CTNNB1: rs1880481 was associated with Karnofsky performance status score, squamous cell carcinoma antigen and Ki-67 proliferation index. Lastly, patients with the AC/AA genotype of CTNNB1: rs1880481 had significantly longer median progression free survival time than those with the CC genotype. In conclusion, SNPs within the Wnt signaling pathway are associated with a decreased risk of bone metastasis, and may be valuable biomarkers for bone metastasis in patients with NSCLC.

## INTRODUCTION

Lung cancer remains the most common malignancy for tumor development and has significantly high cancer-associated mortality and morbidity rates [1]. Non-small cell lung cancer (NSCLC) represents approximately 85% of lung cancer cases [2]. Bone metastasis is observed in approximately 30-40% of patients with NSCLC [3], and it can result in several complications, such as pathological fractures, unbearable bone pain and hypercalcemia crisis [4]. These complications not only reduce the quality of life of the patients but also decrease the survival rate. Moreover, with the current treatment strategies, which included chemotherapy, radiotherapy, targeted therapy and immunotherapy, the mean overall survival time of lung cancer bone metastasis is still only 11.8 months [5]. Therefore, it is particularly important to identify the biological and molecular mechanisms of NSCLC bone metastasis and develop new treatment targets.

Recent studies have shown that a vast majority of molecules participate in the progression of NSCLC bone metastasis [6]. For example, receptor activator of NF-Kb ligand (RANKL) is expressed by osteoblasts and bone marrow stromal cells; it can activate its receptor-RANKL, and result in osteoprotegerin (OPG) binding to regulate the process of local bone resorption by tumor cells [7]. The abnormal function and regulation of the RANKL /RANK /OPG system can result in bone metastasis. In addition, parathyroid hormone-related protein (PTHrP), which is a member of parathyroid hormone family, has been reported to prevent osteoclast apoptosis and promote the growth of bone metastasis [8, 9]. Moreover, a previous study showed that CD147 could induce osteoclast formation and promote bone metastasis in lung cancer by regulating the secretion of interleukin-8 [10]. In clinical research, investigations have demonstrated that patients with younger, have histological subtypes of adenocarcinoma, and have been treated with chemotherapy, radiotherapy, or chemotherapy combined with radiotherapy are independent risk factors for NSCLC bone metastasis [11]. Although many reports focus on the molecular mediators of NSCLC bone metastasis, the underlying mechanism remains unclear.

The Wnt signaling pathway, a well-known pathway, has been reported to be commonly associated with several diseases. Clevers et al. [12] reported that abnormal activation of the Wnt/β-catenin pathway is the initial event and driving force of colorectal cancer. The Wnt signaling pathway is also responsible for the maintenance and proliferation of ovarian cancer stem cells and plays an important role in chemo-resistance to ovarian cancer [13]. Moreover, in NSCLC, studies have shown that the Wnt signaling pathway plays a critical role in maintaining and regulating lung cancer stem cell activity [14]. The Wnt signaling pathway contains classical- and non-classical signaling pathways, and including different components, such as receptors (SFRP2, SFRP4), destruction complex proteins (APC, AXIN, CTNNB1), signaling glycoproteins (WISP3), antagonists (DKK2, DKK3), and transcription factor (GLI-1) [15]. Among them, APC, AXIN1 and CTNNB1 are commonly considered to be the core genes in the canonical Wnt pathway [16]. Furthermore, WNT2 have been reported to promote NSCLC progression and to be strongly associated with poor clinical outcomes in patients with NSCLC [17].

Single nucleotide polymorphisms (SNPs) have been reported to be associated with the development, invasion, and prognosis of lung cancer. A previous study showed that SNPs of multiple genes in the Wnt pathway were associated with the variable clinical prognosis of patients in the early stage of NSCLC [18]. In addition, polymorphisms present in the Wnt signaling pathway were shown to be associated with protective outcome in patients with colorectal cancer in the Saudi population [19]. However, to our knowledge, there is no study that evaluates the potential function of SNPs in the Wnt signaling pathway for NSCLC bone metastasis.

Therefore, in this study, we focused on investigating the relationship between polymorphisms in four core genes in the Wnt signaling pathway, WNT2, AXIN1, CTNNB1 and APC, and the risk of NSCLC bone metastasis. Moreover, we analyzed the association between the significant genotypes and various NSCLC-related indicators, including serum tumor markers, serum calcium, serum phosphorus, the Ki-67 proliferation index, and gene mutation frequency. Through this work, we aim to explore and highlight new prognostic biomarkers for bone metastasis in patients with NSCLC.

## RESULTS

### Clinical characteristics of this study

The clinical characteristics of the 500 patients with NSCLC recruited in this study are described in Table 1. Among these, 308 (61.6%) were male and 192 (38.4%) were female; 37.4% had a history of smoking; 35.4% had KPS > 80; 82.8% had a BMI less than 25 kg/m^2^; and 70.2% were adenocarcinoma lung cancer. According to the AJCC guidelines, 58.4% of patients had stage III or IV. There were 105 patients with bone metastases; 67 of these had bone metastases in the initial diagnosis of NSCLC and the remaining patients developed bone metastases during the follow-up period. In total 54 patients had bone metastases alone and the remaining 51 patients had both bone and other organ metastases (Supplementary Table 1). The median time from diagnosis of NSCLC to bone metastases was approximately 47.5 months.

**Table 1:** Clinical characteristics of non-small cell lung cancer patients and their association with bone metastasis.

**Variable**	**Patients N=500 (%)**	**Events N=105 (%)**	**Univariate analysis**		Multivariate analysis^*^	
			**HR (95% CI)**	***P***	**HR (95% CI)**	***P***
Sex						
Female	192 (38.4)	41 (39.0)	1.000		1.000	
Male	308 (61.6)	64 (61.0)	0.932 (0.629-1.380)	0.724	0.682 (0.395-1.179)	0.170
Age (years)						
≤60	238 (47.6)	46 (43.8)	1.000		1.000	
>60	262 (52.4)	59 (56.2)	1.174 (0.797-1.727)	0.417	1.014 (0.670-1.536)	0.946
Tobacco Smoking Status						
No or Unknown	313 (62.6)	61 (58.1)	1.000		1.000	
Yes	187 (37.4)	44 (41.9)	1.181 (0.801-1.742)	0.401	1.329 (0.793-2.229)	0.281
Body mass index (kg/m^2^)						
<25	414 (82.8)	95 (90.5)	1.000		1.000	
≥25	86 (17.2)	10 (9.5)	0.486 (0.253-0.933)	0.030	0.570 (0.286-1.134)	0.109
Depth of invasion						
Tis, T1, T2	372 (74.4)	58 (55.2)	1.000		1.000	
T3, T4	128 (25.6)	47 (44.8)	2.529 (1.719-3.721)	<0.001	1.260 (0.826-1.922)	0.283
Lymph node metastasis						
N0, N1	295 (59.0)	34 (32.4)	1.000		1.000	
N2, N3	205 (41.0)	71 (67.6)	3.260 (2.161-4.919)	<0.001	1.049 (0.667-1.651)	0.836
Distant metastasis						
M0	293 (58.6)	7 (6.7)	1.000		1.000	
M1	207 (41.4)	98 (93.3)	21.792 (10.107-46.986)	<0.001	10.597 (4.108-27.337)	<0.001
Disease stage at diagnosis						
I, II	208 (41.6)	3 (2.9)	1.000		1.000	
III, IV	292 (58.4)	102 (97.1)	25.024 (7.932-78.942)	<0.001	2.997 (0.705-12.734)	0.137
Tumor histology						
Squamous cell	80 (16.0)	13 (12.4)	1.000		1.000	
Adenocarcinoma	351 (70.2)	76 (72.4)	1.430 (0.793-2.577)	0.234	1.214 (0.655-2.253)	0.538
NSCLC, NOS	69 (13.8)	16 (15.2)	1.613 ((0.775-3.357)	0.201	1.160 (0.547-2.458)	0.699
KPS Score						
>80	177 (35.4)	54 (51.4)	1.000		1.000	
80	281 (56.2)	41 (39.0)	0.757 (0.385-1.487)	0.419	0.591 (0.293-1.193)	0.879
<80	42 (8.4)	10 (9.5)	0.458 (0.305-0.688)	<0.001	1.034 (0.675-1.584)	0.142

^*^Multivariate analyses were adjusted for all the factors listed in this table.

Univariate Cox model analysis revealed that patients with stage III/IV disease (*p* < 0.001), T3/T4 depth of invasion (*p* < 0.001), N2/3 lymph node metastasis (*p* < 0.001) and distant metastasis (*p* < 0.001) had a significantly higher risk of bone metastasis. However, patients with BMI ≥ 25 kg/m^2^ (*p* = 0.030) and KPS < 80 scores (*p* < 0.001) had a lower risk of bone metastasis. For multivariate Cox model analysis, only distant metastasis was significantly associated with bone metastasis (*p* < 0.001). Other clinical variables were not significantly associated with bone metastasis.

### Correlation between the different genotypes of the genes in the Wnt signaling pathway and bone metastasis

**Figure 1:**
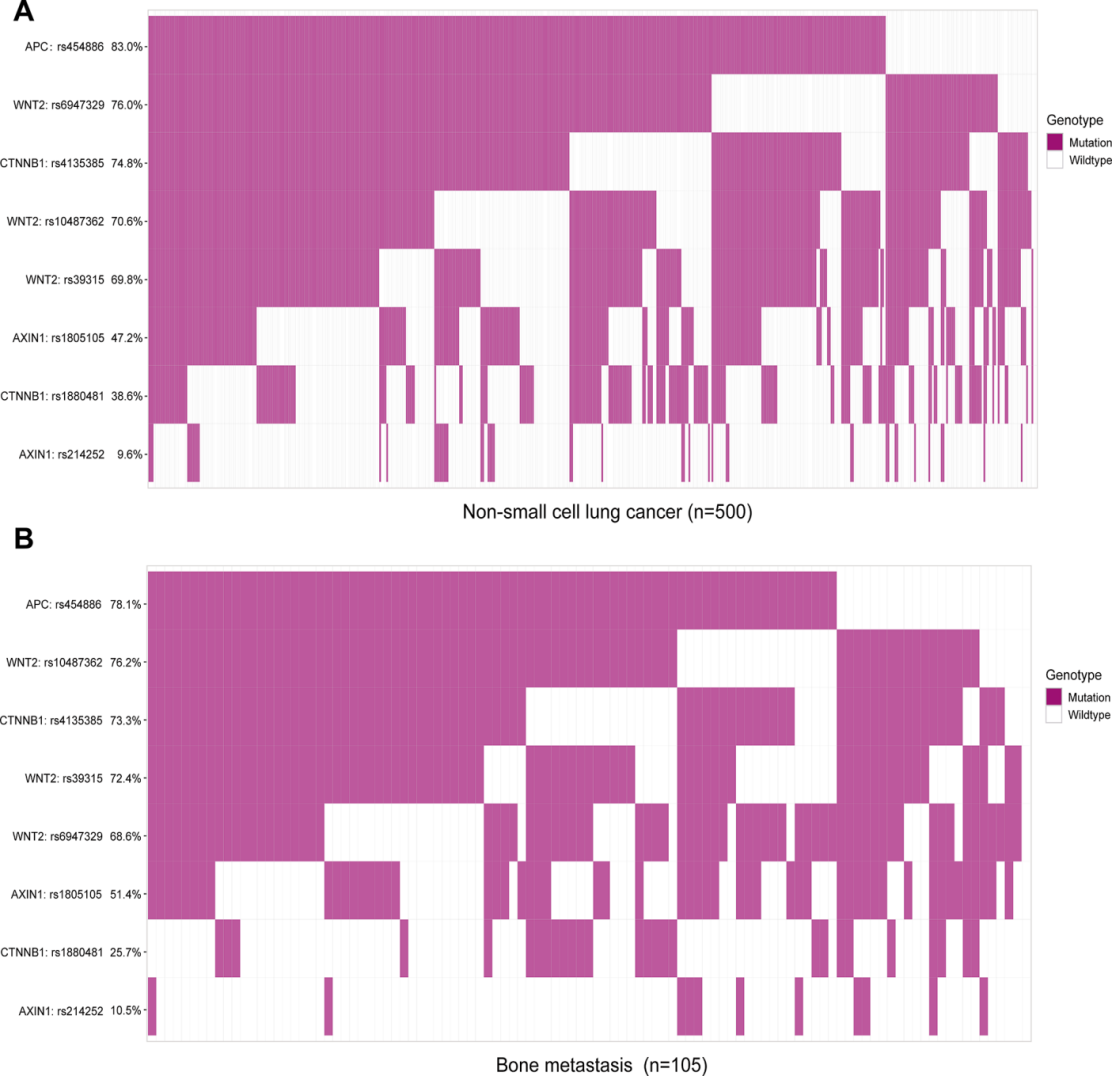
**Mutational frequency rates of eight candidate single nucleotide polymorphisms in patients with non-small cell lung cancer (NSCLC) and bone metastasis.** Classification analysis of SNP genotypes based on mutation and wildtype. (**A**) Analysis of SNPs in all 500 patients with NSCLC, including bone metastasis. (**B**) Analysis of SNPs in 105 patients with bone metastasis from NSCLC.

In total, eight functional SNPs were selected for analysis (Supplementary Table 2): for the WNT2 gene, there were three SNPs (rs10487362 G > A, rs39315 T > C, rs6947329 C > T); for the AXIN1 gene, there were two SNPs (rs1805105 A > G, rs214252 A > G); for the CTNNB1 gene, there were two SNPs (rs1880481 C > A, rs4135385 A > G); and for APC gene, there was only one SNP (rs454886 A > G). Next, we tested the 8 SNPS in 500 NSCLC patients and classified each SNP into either a mutation or wildtype group. As shown in Figure 1, APC: rs454886 had the highest mutation rate among the 500 patients with NSCLC (83%); of these, 105 patients (78.1%) had bone metastases among the 8 candidate SNPs (Figure 1A, 1B). This was followed by WNT2: rs6947329 with 76.0% and 68.6%, respectively, and CTNNB1: rs4135385 with 74.8% and 73.3%, respectively.

**Figure 2:**
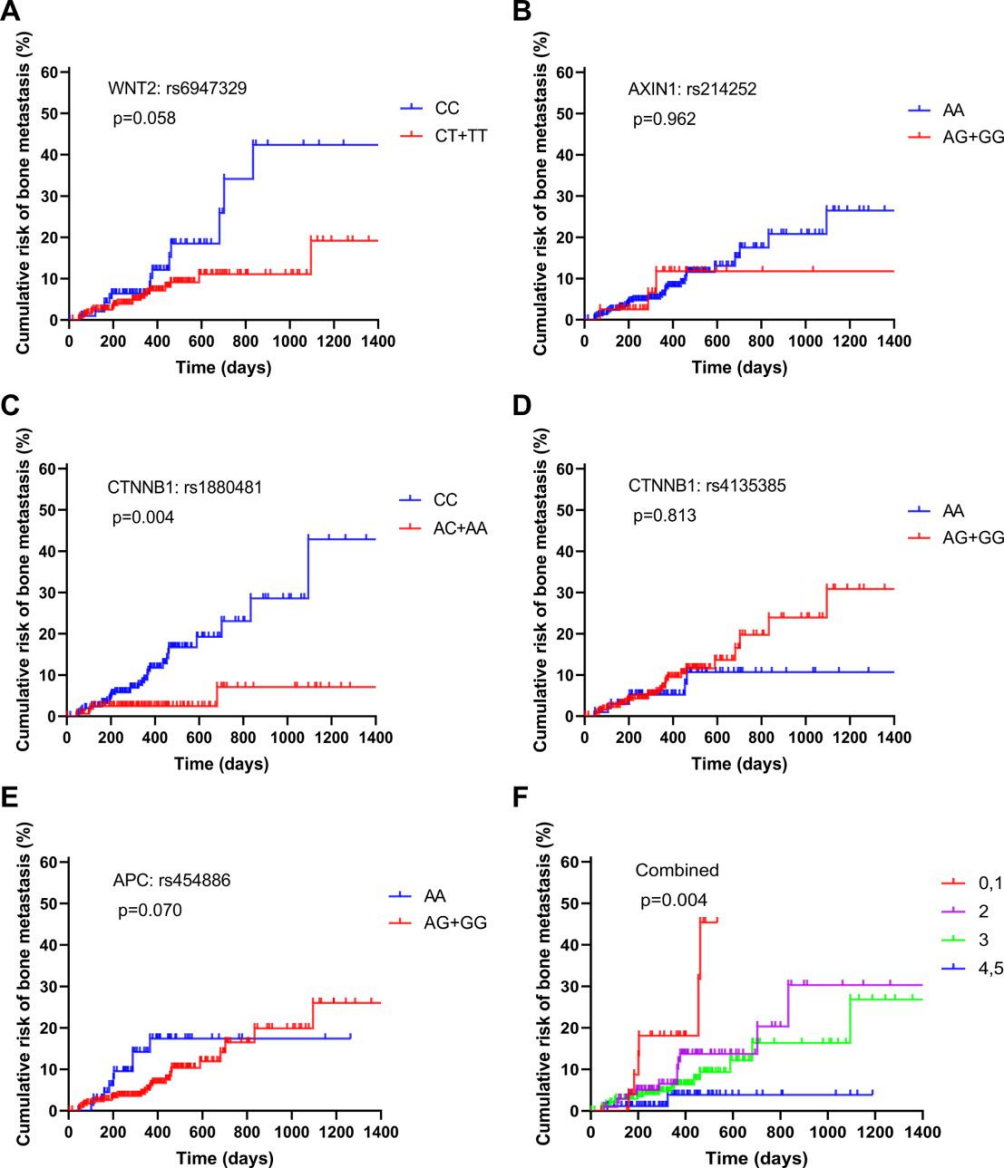
Estimation of the cumulative risk of patients with bone metastasis from NSCLC based on the following protective genotypes: (**A**) WNT2: rs6947329, (**B**) AXIN1: rs214252, (**C**) CTNNB1: rs1880481, (**D**) CTNNB1: rs4135385, (**E**) APC: rs454886, and (**F**) combined.

To detect the impact of these SNPs on prognosis of 500 NSCLC patients, we conducted Kaplan–Meier analysis. Based on our follow-up result, all the 8 SNPs were not significantly associated with the median overall survival (OS) of patients with NSCLC (both *p* > 0.05, Supplementary Figure 1). Then, we attempted to explore the association between the eight Wnt SNPs and the risk of bone metastasis development from NSCLC. Our results revealed that CTNNB1: rs1880481 with the AC/AA genotype was significantly associated with a lower risk of developing bone metastasis (*p* = 0.004, Figure 2C). However, there was no such association in the remaining seven SNPs (Figure 2A, 2B, 2D, 2E).

When taking the distribution of risk genotypes of bone metastasis into account, we found that CTNNB1: rs1880481 with the AC/AA genotype (25.7%, Table 2) had a lower ratio in bone metastasis patients than the CC genotype (74.3%). In addition, we tried to elucidate the relationship between each of the eight SNPs and the risk of bone metastases in NSCLC patients. The univariate Cox model analysis showed that the AC/AA genotype of CTNNB1: rs1880481 was significantly associated with a decreased risk of bone metastasis (HR = 0.729, 95% CI=0.585-0.907, *p* = 0.005). Furthermore, when this univariate Cox model analysis was adjusted for KPS, BMI, disease stage and TNM stage, the result demonstrated that CTNNB1: rs1880481 with the AC/ AA genotype was also significantly correlated with a decreased risk of bone metastasis (HR = 0.742, 95% CI=0.592-0.929, *p* = 0.009). However, there was no association between any of the other seven SNPs and risk of bone metastasis in patients with NSCLC.

**Table 2:** Correlation between different genotypes of genes in Wnt signaling pathway and bone metastasis.

**SNP’s genes**	**Genotypes**	**Patients N=500 (%)**	**Events N=105 (%)**	**Univariate analysis**		**FDR q**	Multivariate analysis^*^		**FDR q**
				**HR (95%CI)**	***P***		**HR (95%CI)**	***P***	
WNT2: rs10487362	GG	147 (29.4)	25 (23.8)	1				1			
	AG+AA	353 (70.6)	80 (76.2)	1.318 (0.841-2.066)	0.229	0.458	1.050 (0.666-1.657)	0.832	0.832
WNT2: rs39315	TT	151 (30.2)	29 (27.6)	1				1			
	CT+CC	349 (69.8)	76 (72.4)	1.047 (0.845-1.297)	0.674	0.763	0.945 (0.759-1.178)	0.616	0.832
WNT2: rs6947329	CC	120 (24.0)	33 (31.4)	1				1			
	CT+TT	380 (76.0)	72 (68.6)	0.828 (0.674-1.018)	0.073	0.243	0.863 (0.700-1.063)	0.165	0.440
AXIN1: rs1805105	AA	264 (52.8)	51 (48.6)	1				1			
	AG+GG	236 (47.2)	54 (51.4)	1.089 (0.899-1.319)	0.385	0.616	1.022 (0.842-1.240)	0.828	0.832
AXIN1: rs214252	AA	452 (90.4)	94 (89.5)	1				1			
	AG+GG	48 (9.6)	11 (10.5)	0.888 (0.475-1.658)	0.709	0.763	0.765 (0.401-1.458)	0.415	0.830
CTNNB1: rs1880481	CC	307 (61.4)	78 (74.3)	1				1			
	AC+AA	193 (38.6)	27 (25.7)	0.729 (0.585-0.907)	0.005	0.040	0.742 (0.592-0.929)	0.009	0.072
CTNNB1: rs4135385	AA	126 (25.2)	28 (26.7)	1				1			
	AG+GG	374 (74.8)	77 (73.3)	0.967 (0.779-1.201)	0.763	0.763	1.031 (0.828-1.284)	0.785	0.832
APC: rs454886	AA	85 (17.0)	23 (21.9)	1				1			
	AG+GG	415 (83.0)	82 (78.1)	0.819 (0.649-1.032)	0.091	0.243	0.841 (0.665-1.063)	0.148	0.440

^*^Multivariate analyses in this table were adjusted for Body mass index, Karnofsky performance status, TNM stage, Disease stage, Abbreviations: HR, hazard ratio; CI, confidence interval.

### Combined analysis of the role of protective SNPs in NSCLC bone metastasis

To explore the combined role of decreased risk SNPs in bone metastasis in NSCLC patients, we defined a genotype with HR of less than 1 as being a “protective” SNP, which included WNT2: rs6947329, AXIN1: rs214252, CTNNB1: rs1880481, CTNNB1: rs4135385 and APC: rs454886. As the number of patients with none or five protective genotypes was small, we combined them with one and four protective genes, respectively, and divided all patients into five groups (Table 3). As a result, bone metastasis developed in 11.5% of patients with none or one protective genotype, in 29.5% of those with two protective genotypes, in 49.5% of those with three protective genotypes, and in 9.5% of those with four or five protective genotypes. Furthermore, the risk of bone metastasis decreased as the number of protective genotypes increased, this indicated that the cumulative role of protective genotypes is “dose-dependent”. The univariate Cox model analysis showed that patients in groups of four or five protective genotypes had a significantly decreased risk of developing bone metastasis (HR = 0.246, 95% CI = 0.106-0.571, *p* = 0.001). In addition, multivariate Cox model analysis, which adjusted for the risk of clinical factors, also confirmed the result of univariate Cox model analysis (HR = 0.293, 95% CI = 0.122-0.702, *p* = 0.006). This accumulative role of protective genotypes for decreased risk of bone metastasis was also confirmed by our Kaplan–Meier analysis (Figure 2F). However, patients in other groups showed no statistical decrease in the risk of bone metastasis.

**Table 3:** Correlation between different protective genotypes and bone metastasis (combined).

**No. of protective genotypes**	**Patients N=500(%)**	**Events N=105(%)**	**Univariate analysis HR (95% CI)**	***P***	**FDR q**	Multivariate analysis^*^ HR (95% CI)	***P***	**FDR q**
0, 1	32 (6.4)	12 (11.5)	1.000			1.000		
2	124 (24.8)	31 (29.5)	0.585 (0.300-1.143)	0.117	0.117	0.587 (0.291-1.183)	0.137	0.137
3	249 (49.8)	52 (49.5)	0.487 (0.259-0.915)	0.025	0.038	0.581 (0.304-1.113)	0.102	0.137
4, 5	95 (19.0)	10(9.5)	0.246 (0.106-0.571)	0.001	0.003	0.293 (0.122-0.702)	0.006	0.018

^*^Multivariate analyses in this table were adjusted for age, sex, smoking status, drinking status, Body mass index, censor, Karnofsky performance status, TNM stage, Disease stage, Abbreviations: HR, hazard ratio; CI, confidence interval.

### Association between the protective SNP and clinical characteristics of NSCLC with bone metastasis

**Figure 3:**
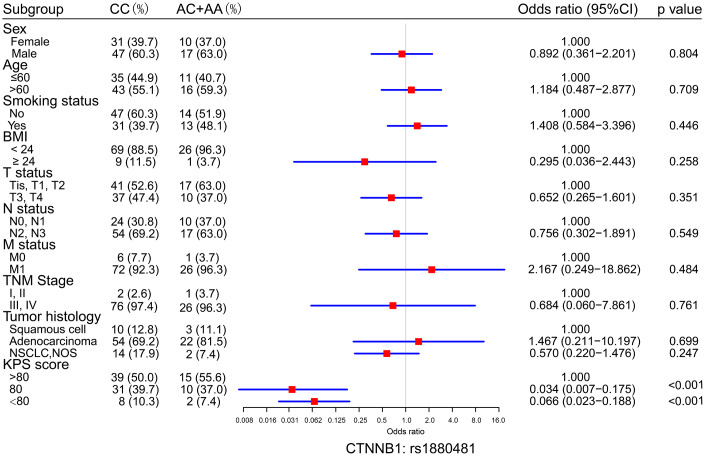
**Logistic regression analysis of the differences in the distribution of clinical characteristics between the CC genotypes and the AC+AA genotypes of CTNNB1: rs1880481.** The odds ratio (OR) with 95% confidence interval (CI) was estimated by logistic regression analysis.

To analyze the association between protective SNPs and NSCLC bone metastasis development, the distribution of clinical characteristics, such as sex, age, smoking status, BMI, T status, N status, M status, clinical stage, tumor histology, and KPS score, in patients with bone metastasis carrying CTNNB1: rs1880481 was valuated. Our results indicated that patients with the AA/AC genotype of CTNNB1: rs1880481 had a lower risk of KPS score down to 80 (OR = 0.034, 95% CI = 0.007-0.175, *p* < 0.001) or less than 80 (OR = 0.066, 95% CI = 0.023-0.188, *p* < 0.001) than those with CC genotype (Figure 3). Other clinical characteristics described above showed no significant differences between the CC and AA/AC genotype of CTNNB1: rs1880481 in patients with NSCLC bone metastasis.

### The protective SNP in association with serum tumor markers in bone metastasis

**Figure 4:**
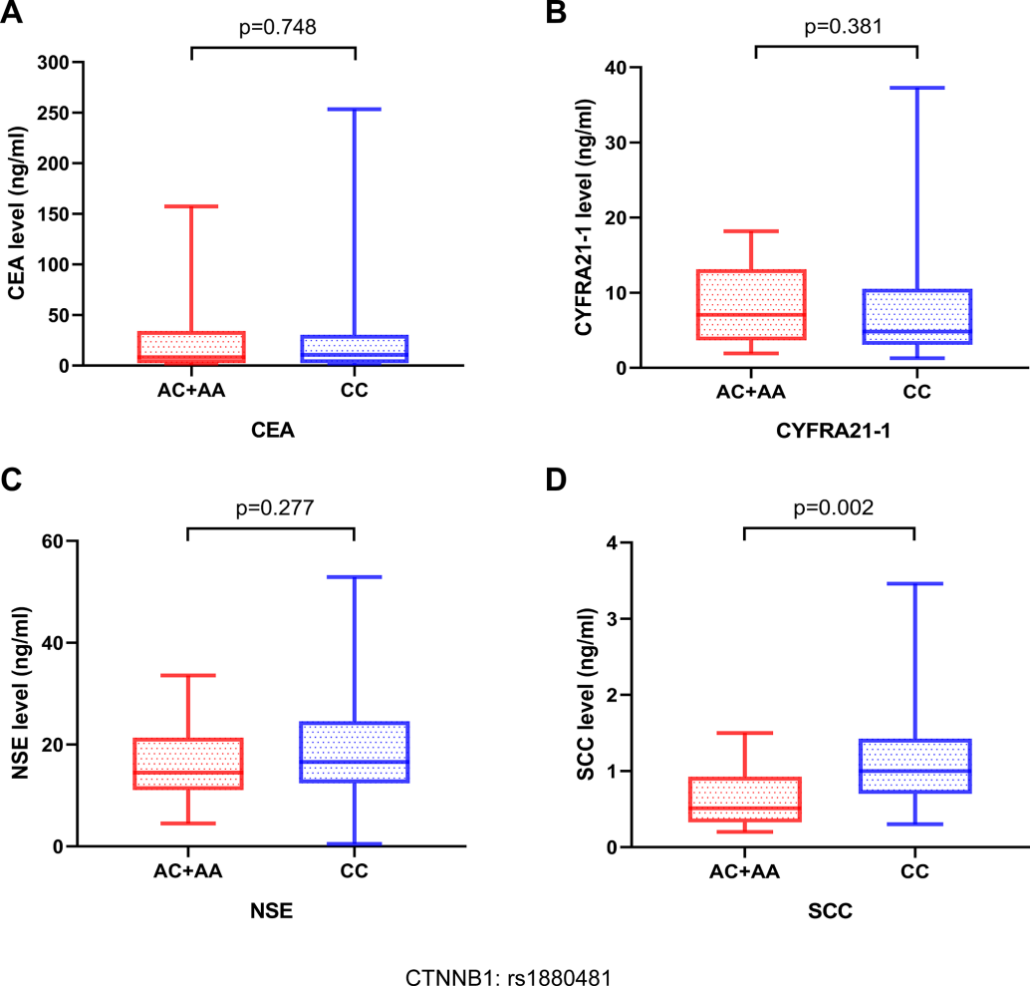
**The relationship between serum tumor markers in patients with NSCLC bone metastases and different genotypes of CTNNB1: rs1880481.** The serum tumor markers contained (**A**) carcinoembryonic antigen (CEA), (**B**) cytokeratin fragment 19 (CYFRA21-1), (**C**) neuron-specific enolase (NSE), and (**D**) squamous cell carcinoma (SCC) antigen.

Carcinoembryonic antigen (CEA), squamous cell carcinoma antigen (SCC-Ag), neuron-specific enolase (NSE), and cytokeratin fragment 19 (CYFRA21-1) are serum tumor biomarkers that play a critical role in the diagnosis and prognosis of lung cancer. Thus, we performed association investigations between the levels of serum tumor markers and SNP of the CTNNB1: rs1880481. Our result demonstrated that patients with bone metastasis in NSCLC carrying the AA/AC genotype of CTNNB1: rs1880481 had a significantly lower level of SCC-Ag than patients with the CC genotype (*p* = 0.002, Figure 4D). For NSE, patients with the AA/AC genotype of CTNNB1: rs1880481 showed a lower median NSE level but no significant difference in comparison with the CC genotype (*p* = 0.277, Figure 4C). Moreover, CTNNB1: rs1880481 carrying the CC genotype and AA / AC genotype had no significant difference in CEA and CYFRA21-1 levels (*p* = 0.748, *p* = 0.381, respectively, Figure 4A, 4B).

### Relationship between the protective SNP and Ki-67 proliferation index, gene mutations, and levels of serum calcium and serum phosphorus in bone metastasis

**Figure 5:**
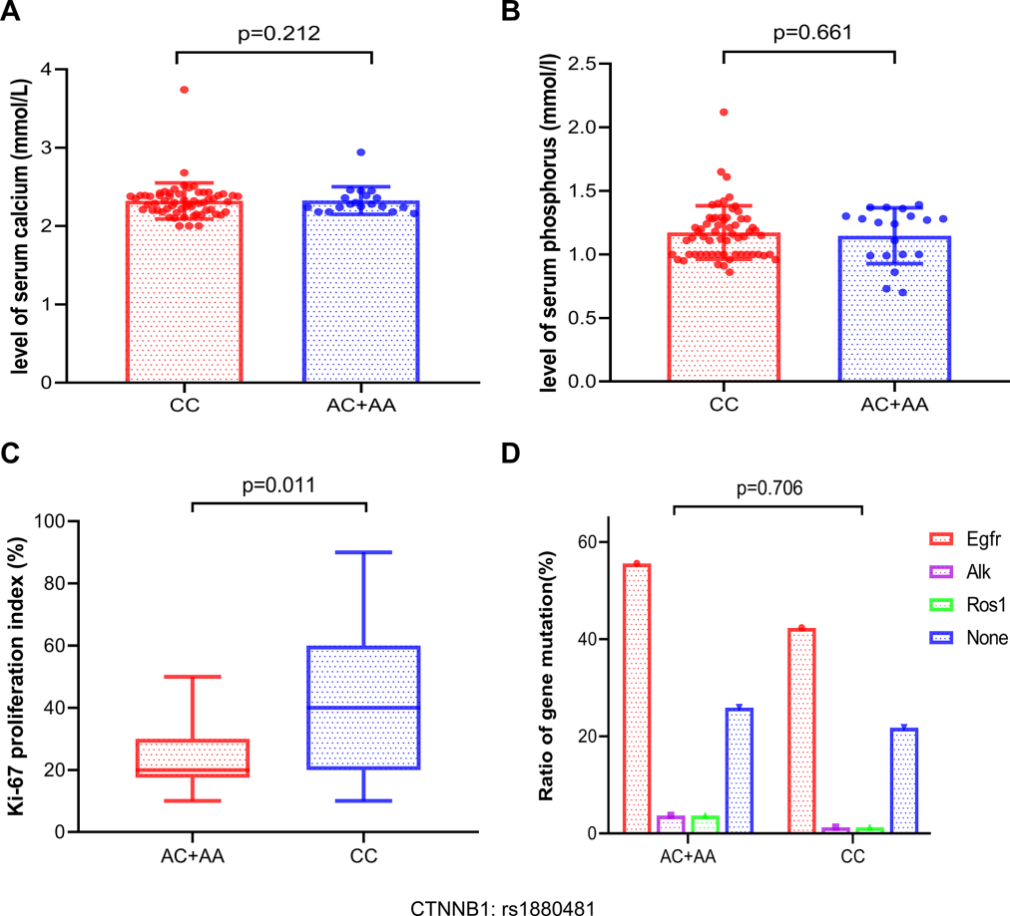
**Analysis of the association between serum calcium, serum phosphorus, ki-67 proliferation index, and gene mutation frequencies and the different genotypes of CTNNB1: rs1880481 in bone metastasis.** (**A**, **B**) The difference of serum calcium and phosphorus levels between the different genotypes. (**C**) Association between with the Ki-67 proliferation index and CTNNB1: rs1880481. (**D**) Analysis of the relationship between gene mutation frequency and the different genotypes of CTNNB1: rs1880481 in patients with NSCLC bone metastasis.

To analyze the relationship between malignant tumor proliferation level and CTNNB1: rs1880481, we collected data on the ki67 proliferation index in 105 NSCLC patients with bone metastasis. Our result showed that patients with bone metastasis in NSCLC carrying the AA/AC genotype of CTNNB1: rs1880481 had a lower level of the Ki-67 proliferation index than those with the CC genotype (*p* = 0.011, Figure 5C). The median Ki-67 proliferation index in patients with AA/AC genotype and CC genotype of CTNNB1: rs1880481 were 30% and 40%, respectively (data not shown). These results indicated that the tumor proliferation level was lower in patients with bone metastasis in NSCLC carrying the AA/AC genotype of CTNNB1: rs1880481.

We also attempted to determine the association between CTNNB1: rs1880481 and the levels of serum calcium and phosphorus in patients with NSCLC bone metastasis; however, no significant differences were observed between them (*p* = 0.212 and *p* = 0.661, respectively, Figure 5A, 5B). Moreover, we attempted to explore the differences between gene mutation patients with bone metastasis in NSCLC and the SNP of CTNNB1: rs1880481. We classified 105 patients with bone metastasis into four groups: EGFR, ALK, ROS1, and none, and the results highlighted that EGFR was the most frequently mutated gene in patients with both the CC genotype (42.3%) and the AA/AC genotype (55.6%) of CTNNB1: rs1880481 (data not shown), compared to the other genes. However, there was no difference in the frequency of all gene mutations between patients with bone metastasis carrying the AA genotype or the AA/AC genotype of CTNNB1: rs1880481 (*p* = 0.706, Figure 5D).

### Impact of protective genotype of SNP on patient prognosis

**Figure 6:**
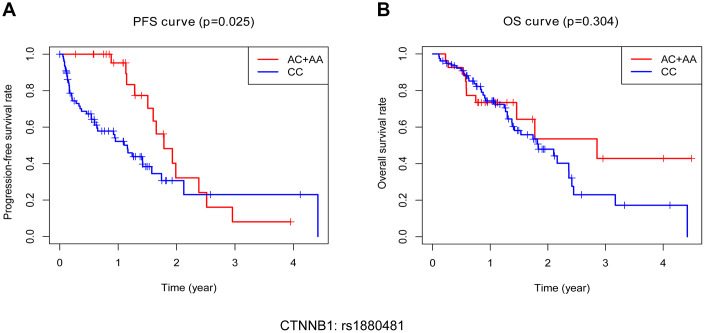
**Kaplan-Meier analysis of progression free survival (PFS) and overall survival (OS) of bone metastasis patients with different genotypes of CTNNB1: rs1880481.** (**A**) PFS of patients with different genotypes. (**B**) OS of patients with different genotypes.

Ultimately, we attempted to explore the impact of protective SNP on patient outcomes, including progression free survival (PFS) and (OS). During a median follow-up of approximately 30 months, a total of 42 NSCLC bone metastasis patients died (40%) and six patients were lost to follow-up (5.7%). According to our follow-up result, patients with the AA/AC genotype of CTNNB1: rs1880481 had a significantly longer median PFS than those with the CC genotype (1.78 years versus 1.16 years, *p* = 0.025, Figure 6A). With regard to OS, however, there was no significant difference found in the median OS between patients with the AA/AC genotype or the CC genotype of CTNNB1: rs1880481 (2.85 years versus 1.83 years, *p* = 0.304, Figure 6B). These results indicated that the AA/AC genotype of CTNNB1: rs1880481 may have an impact on the PFS of patients with bone metastasis in NSCLC, instead of OS.

## DISCUSSION

Globally, NSCLC is one of the most lethal-cancers. Despite the development of various screening techniques and treatment methods, the survival rate of patients with bone metastasis in NSCLC remains generally poor [20]. Therefore, it is essential to identify predictive factors to prevent and improve the survival of patients with bone metastasis in NSCLC. In this study, we analyzed the relationship between eight potential functional polymorphisms of four genes present in the Wnt pathways and the risk of bone metastasis. We found that CTNNB1: rs1880481 is an important protective prognostic factor for NSCLC patients with bone metastases.

Some previous studies demonstrated that SNPs of some genes in the Wnt signaling pathway were associated with clinical outcomes for patients with NSCLC. Coscio et al. [18] reported that rs10898563 of FZD4 gene in the Wnt signaling pathway was correlated with the recurrence risk of patients with early-stage NSCLC. Another recent study confirmed that DKK4: rs2073664 along with three polymorphisms (rs447372, rs419558, and rs17037102) of DKK2 within the Wnt signaling pathway could jointly predict the survival of lung cancer patients who were treated with platinum-based doublet chemotherapy [21]. However, there is no study evaluates the potential roles of SNPs in the Wnt signaling pathway for patients with NSCLC bone metastasis.

In our results, CTNNB1: rs1880481 with the AC/AA genotype significantly reduces the risk of developing bone metastasis, which demonstrates that CTNNB1: rs1880481 is an important SNP associated with the survival of lung cancer, highlighted here for the first time. In recent years, several studies have suggested the significant association between the CTNNB1: rs1880481 and cancer prognosis. A previous study evaluated the relationship between the CTNNB1: rs1880481 polymorphism and gastric cancer; the results demonstrated that CTNNB1: rs1880481 decreased the risk of developing gastric cancer and could be used as a prognostic indicator for the disease [22], which is in accordance with our results. However, there are some studies reporting that CTNNB1: rs1880481 has the opposite effect on other cancers. One study showed that CTNNB1: rs1880481 was associated with poor radiotherapy efficacy in patients with nasopharyngeal carcinoma [23]. In another study, Starinsky et al. [24] reported a noticeable correlation between CTNNB1: rs1880481 and positive familial history of colorectal cancer, suggesting that this SNP was a cancer-promoting factor in colorectal cancer. These conflicting results can have multiple causes, such as the difference in cancer types and genetic susceptibility. Therefore, further studies are warranted to validate our findings.

Previous studies have reported that an increase in the number of protective alleles was associated with better survival rates in cancer. Rebecca et al. [25] reported that non-small-cell lung cancer patients with combined protective alleles of vitamin D receptor (VDR) polymorphisms had improved survival rates. Furthermore, a recent study showed that longer PFS and OS were observed in prostate cancer patients who had more favorable alleles [26]. In our present study, there were five polymorphisms with HR less than 1 showing a cumulative protective impact on the risk of bone metastasis. This indicates that the more protective SNPs, the lower risk of NSCLC bone metastasis. Our results are consistent with those of previously reported studies.

Furthermore, there are a number of clinical characteristics that have been shown to be associated with bone metastasis in patients with lung cancer [5, 27]. A recent report proposed that N3 staging showed a higher risk of bone metastasis compared to other N staging [28]. Another study reported that the risk of lung cancer bone metastasis was related to younger age, histological types of adenocarcinoma and some therapeutic interventions [11]. However, our study showed that SNP of CTNNB1: rs1880481 was associated with a lower risk of KPS score down to 80 or less than 80, not with N staging or other clinical characteristics. The contrasting results may be due to different clinical factors of the sample as well as sample size. Nonetheless, our results suggest that CTNNB1: rs1880481 is a protective factor for patients with bone metastases in NSCLC.

Ki67 has been shown to be associated with cancer metastasis. Green et al. [29] have shown that high Ki67 expression is associated with shortened survival and increased risk of metastasis in prostate cancer. Moreover, overexpression of Ki67 is considered to be related to the development of bone metastasis in breast cancer patients [30]. In our study, we found that bone metastasis patients with CTNNB1: rs1880481 AC/AA genotype had a significantly lower Ki-67 index than those with the CC genotype. This suggests that CTNNB1 polymorphic variants may be a protective prognostic biomarker for NSCLC bone metastasis. Coincidentally, we observed that NSCLC bone metastasis patients with the AC/AA genotype of CTNNB1: rs1880481 have a longer PFS, which further indicates CTNNB1: rs1880481 may be a protective prognostic biomarker. However, there was no significant difference in OS between patients with the AC/AA genotype or the CC genotype of CTNNB1: rs1880481. Therefore, further studies are required to fully explore the effect of CTNNB1: rs1880481 on bone metastasis in NSCLC.

Several studies have previously reported that serum calcium or serum phosphorus is associated with bone metastasis [28]. Shen et al. [31] have shown that low serum calcium levels increase the risk of bone metastasis in NSCLC patients. However, in our study, the bone metastasis protective factor- CTNNB1: rs1880481 had no significant relationship with serum calcium and phosphorus levels. This discrepancy may be due to the different sample sizes or clinical heterogeneity of included subjects, and so, further studies need to be conducted. NSCLC patients with EGFR mutation has been reported to be a controversial prognostic factor for bone metastases. For example, studies have revealed that patients with EGFR mutation have been investigated and considered to be more likely to develop bone metastases and also have a poorer prognosis [20, 32]. However, Hendriks et al. [33] reported that there was no noteworthy difference between EGFR, KRAS, WT mutation and bone metastases. As for our study, the frequency mutations of EGFR, ROS1, and EML-ALK were not different in patients with the AC/AA genotype of CTNNB1: rs1880481 and those with the CC genotype. These results indicate that the association between gene mutations and outcomes of NSCLC bone metastasis requires more in-depth studies.

Serum tumor biomarkers including CEA, SCC-Ag, NSE and CYFRA21-1 are known to be prognostic or predictive biomarkers in lung cancer. Among them, SCC-Ag is a specific marker for lung squamous cell carcinoma (SCC) and plays an important role in NSCLC [34]. In the present study, we found that patients with the AA/AC genotype of CTNNB1 : rs 1880481 had lower levels of SCC-Ag and longer PFS. A previous study has reported that advanced NSLCL patients with mutant-type EGFR had lower levels of SCC-Ag and longer PFS than those with wild-type EGFR [35]. These results indicated that the effect of EGFR mutation on advanced NSCLC was consistent with the impact of CTNNB1:rs1880481 on bone metastasis of NSCLC in our study. Additionally, a recent study showed that patients with SCC type of NSCLC bone metastases had a poor survival outcome [36]. These studies further validated that CTNNB1: rs1880481 may be a potential protective biomarker for bone metastasis in NSCLC patients. Some investigations demonstrated that levels of serum CEA and NSE, instead of CYFRA21-1, were significantly higher in NSCLC patients with bone metastases than those without bone metastases [37, 38]. However, we have not found the relationship between CTNNB1: rs1880481 and the three serum tumor biomarkers, indicating that a multi-center study and sufficient clinical data are necessary to verify the results.

Nonetheless, there are some limitations in our present study. Firstly, our research was only carried out in one institution. Additionally, we performed multiple analytical techniques evaluating the relationship between clinical features and bone metastasis-related SNPs, which may cause false-positive results in data analysis. Lastly, the molecular mechanism underlying the effect of CTNNB1: rs1880481 on bone metastasis of lung cancer remains unclear. Therefore, further in-depth studies are needed to confirm our findings.

In conclusion, and based on the findings presented in this study, we conclude that SNPs within the Wnt signaling pathway are associated with the decreased risk of bone metastasis and may be independent protective factors and valuable biomarkers for patients with NSCLC bone metastasis.

## MATERIALS AND METHODS

### Study population

In this study, we recruited 540 unrelated patients with NSCLC of Han Chinese descent from Fujian Provincial Hospital. The patients were newly diagnosed and confirmed to have NSCLC by pathology between May 2015 and October 2017. There were no restrictions on sex, age, smoking status, body mass index (BMI), Karnofsky performance status (KPS), histology, or disease stage. Patients were required to provide sufficient blood samples for genotype analysis. Patients with other cancers or cases in which the tumor origins were uncertain were excluded from the study.

Epidemiological information, including sex, age, smoking history, BMI, medical history, potential carcinogen exposure history, and family history of cancer, was collected through a structured questionnaire given to the patients. Clinical and pathological data, including gene mutation status, treatment regimens, and TNM staging data were collected from medical records. Patient staging was based on the 8^th^ edition manual of the American Joint Commission on Cancer (AJCC) staging system. The diagnosis of bone metastasis was based on emission computed tomography scans.

Patients underwent outpatient service or telephone call follow-up every 2-3 months, and survival information of each patient was acquired from the follow-up records. The last follow-up time was November 2019.

Of all patients recruited in this study, 40 patients were excluded: 20 were due to the inability to gain information on bone metastasis, 19 owing to incomplete data on TNM staging, and 1 for failed genotyping. The remaining 500 patients were enrolled for analysis.

All participating patients signed informed consent forms, and this study was approved by the Medical Ethics Committee of Fujian Provincial Hospital (K2017-11-006).

### SNP selection and genotyping

The public NCBI dbSNP database (http://www.ncbi.nlm.nih.gov/), HapMap SNP database (https://www.broadinstitute.org/medical-and-population-genetics/hapmap-3) and SNPinfo database (https://snpinfo.niehs.nih.gov/) were used to select the potential functional SNPs in the four core genes of the Wnt signaling pathway, WNT2, AXIN1, CTNNB1 and APC, present in the Chinese Han population. The selection of SNPs was based on the following criteria: 1) linkage disequilibrium coefficient r^2^ < 0.8; 2) minor allele frequency (MAF) ≥ 5%; 3) located in the genes’ regulatory region; and 4) effects on the action of transcription factor binding site or microRNA binding site.

### Genomic DNA isolation and genotype analysis

The EasyPure Blood Genomic DNA kit (EE121, TransGen Biotech) was used to extract the genomic DNA from peripheral blood samples based on the manufacturer’s instructions. Then, we designed corresponding primers for the selected eight SNPs (Supplementary Table 3) and used the matrix- assisted laser desorption/ionization-time-of-flight mass spectrophotometry (MALDI-TOF-MS) to obtain allele-specific primer extension products. Finally, we used the MassARRAY platform (Agena Bioscience) and Sequenom TYPER software (version 4.1) to analyze the sequencing data. A blind and randomized analysis of 5% genomic DNA samples was performed to assess the reproducibility, and our results revealed reproducibility rates of 99%.

### Statistical analysis

We used the Kaplan-Meier test to assess the difference in survival time between patients with bone metastasis and those without bone metastasis. We conducted Cox proportional hazards regression analysis to evaluate the effects of genotype on the risk of developing bone metastasis. The Cox regression analysis was adjusted for sex, age, smoking status, BMI, KPS, tumor histology, and TNM staging. To plot the cumulative protective risk of bone metastasis, we performed Kaplan-Meier survival curves. To make multiple comparison correction, we used the R-package to computed the q-value (a false discovery rate [FDR] adjusted P-value). We used the χ^2^ test to investigate the discrepancy of gene mutation frequency between two groups and Student’s *t* test to compare the distinction of serum tumor markers, serum calcium, serum phosphorus and the Ki-67 proliferation index between the groups. Statistical analysis was conducted using SPSS version 20.0 software, and *p* values of less than 0.05 were considered statistically significant.

### Ethical statements

All patients signed a written informed consent for blood sample collection and data, and the study was approved by the Medical Ethics Committee of Fujian Provincial Hospital, Fuzhou, China.

## Supplementary Material

Supplementary Figure 1

Supplementary Tables
