# Supplementary material for: Single nucleotide polymorphisms within the Wnt pathway predict the risk of bone metastasis in patients with non-small cell lung cancer
Source: Aging (Albany NY). 2020 May 26;12(10):9311–27. doi: 10.18632/aging.103207 (PMC7288946; doi:10.18632/aging.103207)
Supplement: Supplementary Figure 1 [file aging-12-103207-s002..pdf]

SUPPLEMENTARY FIGURE

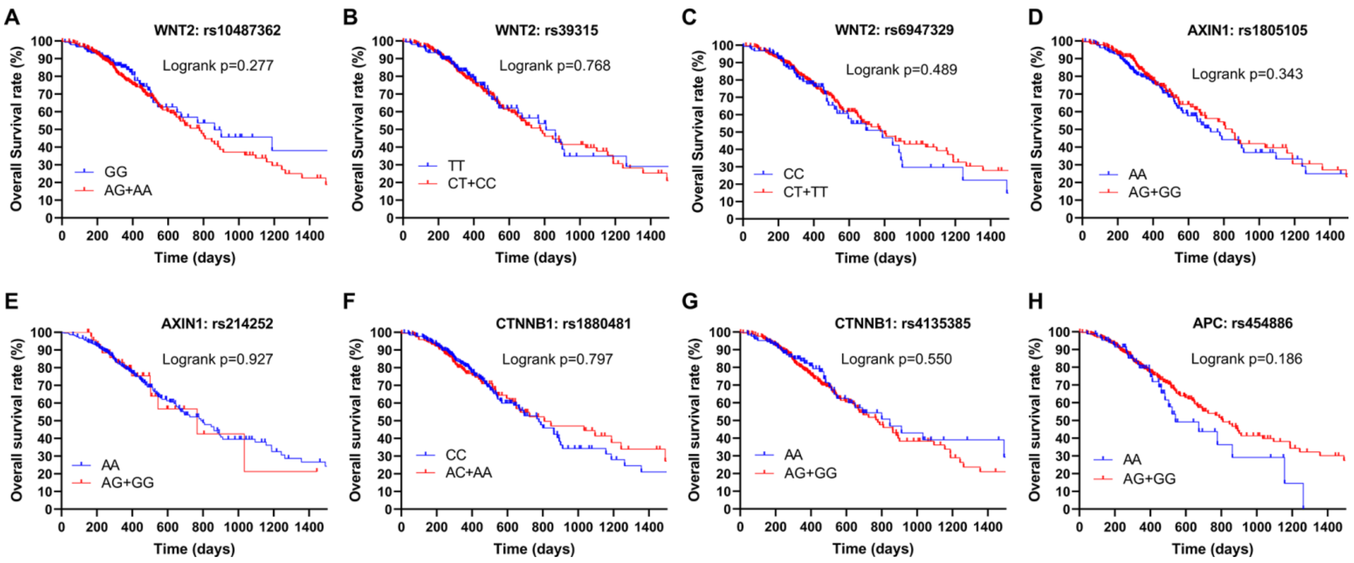

Supplementary Figure 1. Evaluation of the overall survival rate of NSCLC patients according to the following genotypes: (**A**) WNT2: rs10487362, (**B**) WNT2: rs39315, (**C**) WNT2: rs6947329, (**D**) AXIN1: rs1805105, (**E**) AXIN1: rs214252, (**F**) CTNNB1: rs1880481, (**G**) CTNNB1: rs4135385, (**H**) APC: rs454886.
