# Supplementary material for: Single nucleotide polymorphisms within the Wnt pathway predict the risk of bone metastasis in patients with non-small cell lung cancer
Source: Aging (Albany NY). 2020 May 26;12(10):9311–27. doi: 10.18632/aging.103207 (PMC7288946; doi:10.18632/aging.103207)
Supplement: Supplementary Tables [file aging-12-103207-s001..pdf]

SUPPLEMENTARY TABLES

Supplementary Table 1. Distribution of metastatic sites in patients with NSCLC bone metastases.

Metastasis site	N=105(%)
Bone metastases alone	54 (51.43)
Bone + Liver	6 (5.71)
Bone + Brain	23 (21.90)
Bone + Adrenal gland	7 (6.67)
Bone + Liver + Adrenal gland	6 (5.71)
Bone + Liver + Brain	5 (4.76)
Bone + Liver + Brain + Adrenal gland	2 (1.91)
Bone + Brain + Adrenal gland	2 (1.91)

Supplementary Table 2. SNP information of genes selected in this study.

Gene	Polymorphic site	Allelic change	SNP position	Chromosome	MAF[a]
WNT2	rs10487362	G>A	Intron	Chr7	0.23
	rs39315	T>C	Promoter	Chr7	0.47
	rs6947329	C > T	Intron	Chr7	0.45
AXIN1	rs1805105	A>G	Coding exon	Chr16	0.36
	rs214252	A>G	Downstream	Chr16	0.21
CTNNB1	rs1880481	C>A	Intron	Chr3	0.35
	rs4135385	A>G	Intron	Chr3	0.23
APC	rs454886	A>G	Intron	Chr5	0.35

[a]MAF: Minor Allele Frequency.

Supplementary Table 3. Primer for selected SNPs in genotyping.

Gene and polymorphisms	Forward primer	Reverse primer
WNT2: rs10487362	ACGTTGGATGGTGAGTGAAAAATGCCCCTC	ACGTTGGATGACGTGAACTAATGCATCAGC
WNT2: rs39315	ACGTTGGATGAACCTTTAGGGAAGCTCCAG	ACGTTGGATGTCAGGGACTTGTTTGCCAGC
WNT2: rs6947329	ACGTTGGATGGATCATTTCCAGCCTCTCCC	ACGTTGGATGACCACAAGTACCTTCAAGGC
AXIN1: rs1805105	ACGTTGGATGTGTCTCCAGGAGCAGCTTC	ACGTTGGATGTGAGGAATGGAAGTGTGACC
AXIN1: rs214252	ACGTTGGATGCGTGGCGTGCAAAAGAAATG	ACGTTGGATGTTCTGGTTCTTCTCCGCATC
CTNNB1:rs1880481	ACGTTGGATGTCCCCGTGCTTAATTCTTAG	ACGTTGGATGGCTGTCATTTCTCCCATAAC
CTNNB1: rs4135385	ACGTTGGATGACATGCACAAAGCAAGGAAG	ACGTTGGATGGTTGGAGTTACTTGTTCCTT
APC: rs454886	ACGTTGGATGGTCAGCTCTCCTTTCTTTGG	ACGTTGGATGGAAGGTAAAGTTATATGCAG
